# PNS-R1 inhibits Dex-induced bronchial epithelial cells apoptosis in asthma through mitochondrial apoptotic pathway

**DOI:** 10.1186/s13578-019-0279-x

**Published:** 2019-02-25

**Authors:** Wenjing Zou, Chao Niu, Zhou Fu, Caihui Gong

**Affiliations:** 1 Department of Respiratory Medicine, Children’s Hospital of Chongqing Medical University, Ministry of Education Key Laboratory of Child Development and Disorders, Chongqing, 400014 China; 2 China International Science and Technology Cooperation base of Child Development and Critical Disorders, Chongqing Key Laboratory of Pediatrics, Chongqing, 400014 China

**Keywords:** PNS-R1, Dex, Apoptosis, Asthma, Bronchial epithelial cells

## Abstract

Dexamethasone (Dex) are widely used for the treatment of asthma. However, they may cause apoptosis of bronchial epithelial cells and delay the recovery of asthma. Therefore, it is an urgent problem to find effective drugs to reduce this side effects. Panax notoginseng saponins R1 (PNS-R1) is known to exhibit anti-oxidative and anti-apoptotic properties in many diseases. We aim to investigate whether PNS-R1 can reduce Dex-induced apoptosis in bronchial epithelial cells. In this study, the anti-apoptotic effects of PNS-R1 were investigated by conducting in vitro and in vivo. Annexin V-FITC/PI staining flow cytometry analysis and TUNEL assay were conducted to detect apoptotic cells. Mitochondrial membrane potential was detected by JC-1 analysis. Western blotting and immunohistochemical analysis were conducted to measure caspase3, Bcl-2, Bax, Cyt-c, Apaf-1, cleaved-caspase3 and cleaved-caspase9 levels in lung tissues and 16HBE cells. Our findings demonstrated that Dex could induce apoptosis of bronchial epithelial cells and upregulate caspase3 expression of lung tissues. Western blot showed that Dex increased Bax, Cyt-c, Apaf-1, cleaved-caspase9, cleaved-caspase3 expression and decreased Bcl-2 expression. PNS-R1 could suppress Dex-induced apoptosis of bronchial epithelial cells by inhibiting Bax, Cyt-c, Apaf-1, cleaved-caspase9, cleaved-caspase3 expression and upregulating Bcl-2 expression. Flow cytometry analysis showed PNS-R1 alleviated JC-1 positive cells induced by Dex in 16HBE cells. These results showed that PNS-R1 alleviated Dex-induced apoptosis in bronchial epithelial cells by inhibition of mitochondrial apoptosis pathway. Furthermore, our findings highlighted the potential use of PNS-R1 as an adjuvant drug to treat asthma.

## Introduction

Asthma is one of the most common chronic diseases worldwide-an increasing tendency of the incidence [1]. Inhaled corticosteroids (ICS) are the most commonly used drugs for the treatment of asthma [2]. However, Dex therapy in asthma can induce airway epithelial cell apoptosis and inhibit cell proliferation, which in turn inhibit epithelial repair and lead to airway remodeling [3]. Some studies also suggested that the use of inhaled corticosteroids could affect airway remodeling and epithelial damage in many patients with chronic and persistent asthma [4]. Therefore, there is an urgent need to explore the mechanisms underlying glucocorticoid-induced apoptosis in airway epithelial cells in asthma and to develop therapeutic strategies for asthma.

Panax notoginseng saponins R1 (PNS-R1), which is an important component of the Chinese medicine *Sanqi*, is known for its anti-inflammatory, anti-oxidative and anti-apoptotic properties [5]. Previous studies have demonstrated that PNS-R1 exerted protective effects against ischemia-induced apoptosis in vitro and in vivo models of cardiomyocytes [6]. PNS-R1 has been shown to promote angiogenesis in human umbilical vein endothelial cells [7]. PNS-R1 could inhibit PC12 cell apoptosis induced by oxidative injury [8]. But whether PNS-R1 could reduce Dex-induced apoptosis of bronchial epithelial cells and its specific mechanism are unknown.

Mitochondrial pathway is an important endogenous pathway of apoptosis. Mitochondrial permabilization leads to the formation of an apoptosome, which facilitates caspase activation and subsequently triggers the other proteins of apoptotic cell death.  The mitochondria-dependent pathway involving Bcl-2 and Bax. Activation of Bax leads to the release of many mitochondrial proteins, such as cytochrome c (Cyt-c) via translocation of Bax from the cytosol to mitochondria, overcoming the regulation by Bcl-2 of mitochondrial membrane protein permeability.  An apoptosome complex then forms through the binding of Cyt-c, apoptotic, protease, activating factor 1 (Apaf-1), procaspase9, and dATP. Then the apoptosome complex dimer activates caspase9 and further activates caspase3, which degrade to induce apoptosis [9]. A study showed that notoginsenoside R1 inhibited the increased number of cells positive to propidium iodide (PI) staining and depolarization of mitochondrial membrane potential in cultured neurons exposed to glutamate, in addition to blocking decreased Bcl-2 and increased Bax expression levels [10]. Notoginsenoside R1 also attenuated endoplasmic reticulum stress response and neuronal apoptosis, caspase12 was increased, and Bcl-2 was decreased [11]. Notoginsenoside R1 inhibited apoptosis by inhibiting mitochondrial membrane potential disruption, caspase3 activation, and DNA fragmentation for cerebral ischemia–reperfusion (I/R) injury [12]. Therefore, we speculated whether PNS-R1 could reduce Dex-induced apoptosis of mitochondrial apoptosis of bronchial epithelial cells in asthma through mitochondrial apoptosis pathway.

## Materials and methods

### Animals and treatments

Female C57BL/6 mice (6 to 8 weeks old) were purchased from the Experimental Animal Center of Chongqing Medical University (Chongqing, China). The mice were housed under specific pathogen-free conditions and subjected to a 12 h/12 h dark/light cycle. The study was approved by the Ethics Committee of Chongqing Medical University. A total of 40 mice were divided into five groups, including control group, asthma group, Dex group, PNS-R1 group and Dex + PNS-R1 group (n = 8 in each group). To induce asthma, mice were sensitized to 20 μg of house dust mite (HDM; Greer, Los Angeles, CA, USA) in 30 μl normal saline (NS) via nasal inhalation on days 0, 14, 21, 23, 25, 27 and 29 [13]. The control mice were similarly treated with NS. On the last 3 days, mice in the PNS-R1 group were administered 5 mg/kg PNS-R1 (ip) (Macklin, Shanghai, China) [14, 15] at 30 min before HDM inhalation. Mice in the Dex group were administered with 5 mg/kg Dex (ip) (Axxora, Farmingdale, NY, USA) at 30 min before HDM inhalation [16]. While mice in the PNS-R1 + Dex group were administered 5 mg/kg PNS-R1 (ip) and 5 mg/kg Dex (ip) at 30 min before HDM inhalation. All mice were sacrificed at 24 h after the last HDM inhalation and then euthanized by intraperitoneal injection of 4% pentobarbital (0.5 mg/kg).

### Cell culture and treatments

Human bronchial epithelial cells (16HBE) were obtained from the American Type Culture Collection (ATCC, USA) and cultured in Dulbecco’s Phosphate Buffered Saline medium (DMEM) containing 10% fetal bovine serum (FBS; Gibco, USA). The cells were cultured at 37 °C in 5% CO_2_ atmosphere, and the growth status of cells was evaluated under a microscope. Cells were grown to 85% to 90% confluence, cells passaged, and subjected to trypsin digestion. Cells were divided into control group (16HBE), Dex group (Dex), PNS-R1 group (PNS-R1) and Dex + PNS-R1 group (Dex + PNS-R1). Cells in Dex group were treated by Dex (5 μM) [17] and PNS-R1 group were treated by PNS-R1 (2 μM) [14] for 24 h. The Dex + PNS-R1 group cells were treated by both Dex (5 μM) and PNS-R1 (2 μM).

### Immunohistochemical (IHC) staining

Left lung tissues of mice were fixed in 4% formalin buffer and embedded in paraffin. The paraffin blocks were then serially sectioned into 4-μm-thick slices and subjected to IHC staining. The sections were deparaffinized in xylene for 25 min and rehydrated in ethanol for 5 min. The sections were incubated in 3% H_2_O_2_ for 10 min; 10 mM sodium citrate buffer was used for antigen retrieval in a microwave oven for 3 min at high power and 15 min at low power. The sections were then blocked in goat serum (Wuhan Boster Biological Technology, Wuhan, China) for 30 min at room temperature. Rabbit antibody against caspase3 (1:500; Proteintech, Wuhan, China) was used for IHC staining overnight at 4 °C, followed by a 30 min incubation with a secondary goat anti-rabbit antibody at 37 °C. The sections were washed in PBS and incubated with 3,3-diami-nobenzidine (DAB, Zhongshan Golden Bridge, Beijing, China) for 3 min. Slides were counterstained with hematoxylin before microscopic analysis.

### Microscope and image analysis

The caspase3 expression in lung tissues was subjected to microscopic analysis. Briefly, after IHC staining, if a cell or tissue was stained from light yellow to brown, it would be recorded as positive immunostaining. The areas from bronchial epithelial cells were selected for analysis. The intensity of the staining signal was measured and documented using the Image-Pro Plus 6.0 image analysis software (Media Cybernetics, Inc. Silver Spring, MD USA). The mean densitometry of the digital image (×200) is designated as representative integral optical density (IOD)/area staining intensity (indicating average optical density analysis). The signal density of tissue areas from three randomly selected visions of slice 3–6 different animal tissues in each group were counted blindly and subjected for statistical analysis.

### Western blot analysis

The proteins in each right lung tissue and 16HBE cells were isolated using a total protein extraction kit (KeyGen BioTECH, Jiangsu, China). Protein concentration was determined by conducting a bicinchoninic acid assay following standard techniques. Protein lysis samples (30 µg) were resolved by 10% sodium dodecyl sulfate polyacrylamide gel electrophoresis (SDS-PAGE). Afterwards the samples were transferred onto polyvinylidene fluoride (PVDF) membranes. Membranes were then blocked and incubated with the primary antibody 4 °C overnight, and subsequently incubated with the secondary goat anti-rabbit antibody (1:2000; ProteinTech, Wuhan, China). The primary antibodies includes rabbit anti-caspase3 (1:500; ProteinTech, China), rabbit anti-caspase9 (1:500; ProteinTech, China), rabbit anti-Bcl2 (1:200; Abcam, UK), rabbit anti-Bax (1:200; Abcam, UK), rabbit anti-Cyt-c (1:500; CST, USA), rabbit anti-Apaf-1 (1:500; CST, USA), rabbit anti-cleaved-caspase9 (1:500; ProteinTech, China), rabbit anti-cleaved-caspase3 (1:500; ProteinTech, China) and rabbit anti-β-actin antibodies (1:1000; ProteinTech, Wuhan, China). Band intensities were quantified using Quantity One software (version 4.6.2; Bio-Rad, Hercules, CA, USA) and normalized relative to that of the inner control.

### Flow cytometry

To detect cell apoptosis, 16HBE cells were seeded in six-well plates (Corning, NY, USA) and divided into the following four treatment groups: PNS-R1 + Dex group, PNS-R1 group, Dex group and control group. Cells were treated with Dex (5 μM) and PNS-R1 (2 μM) for 24 h. Apoptosis was measured by flow cytometry using the Annexin V-PI apoptosis-detection kit (KeyGen BioTech, China) according to the manufacturer’s instructions. To detect the cell mitochondrial membrane potential (Δψm), we also used the JC-1 apoptosis-detection kit (KeyGen BioTech, China). The percentage of apoptotic cells was calculated using the FASC Calibur MT flow cytometer (BD Bioscience, NJ, USA).

### TUNEL assay

TUNEL assay was performed to detect the apoptotic tracheal epithelial cells in the lung tissues and 16HBE cells using the InSitu Cell Death Detection kit (Roche, Switzerland) according to the manufacturer’s instructions. The nucleus is counterstained with DAPI fluorescent dye (Beyotime BioTech, China) for total cell count. A fluorescence microscope (Olympus, Tokyo, Japan) was used to obtain the images and assess the apoptotic cells. The TUNEL positive cells in each slice were calculated by randomly selecting three different fields. Three fields of view were randomly selected for each cell slide, and 3–6 pieces of each group were taken. ImageJ software is used to count the number of cells in pictures and the percentage of apoptotic cells subjected for statistical analysis.

### Statistical analyses

Data were expressed as mean ± standard error of the mean. GraphPad Prism software (version 5.0; GraphPad, San Diego, CA, USA) was used for statistical analyses. Two-way analysis of variance was conducted to determine statistically significant differences in the tested variables among the different groups. If an overall test was significant, Tukey’s test was used for specific comparisons between groups. P < 0.05 were considered statistically significant. All experiments were repeated at least thrice with consistent outcomes.

## Results

In our previous study [16, 17], we showed that Dex inhibited bronchial epithelial cell proliferation and migration in a dose- and time-dependent manner. The dose and time of Dex treatment used in our subsequent experiments were based on the procedures in our previous study.

### PNS-R1 reduces Dex-induced caspase3 protein expression of bronchial epithelial cells in asthmatic mice

**Fig. 1:**
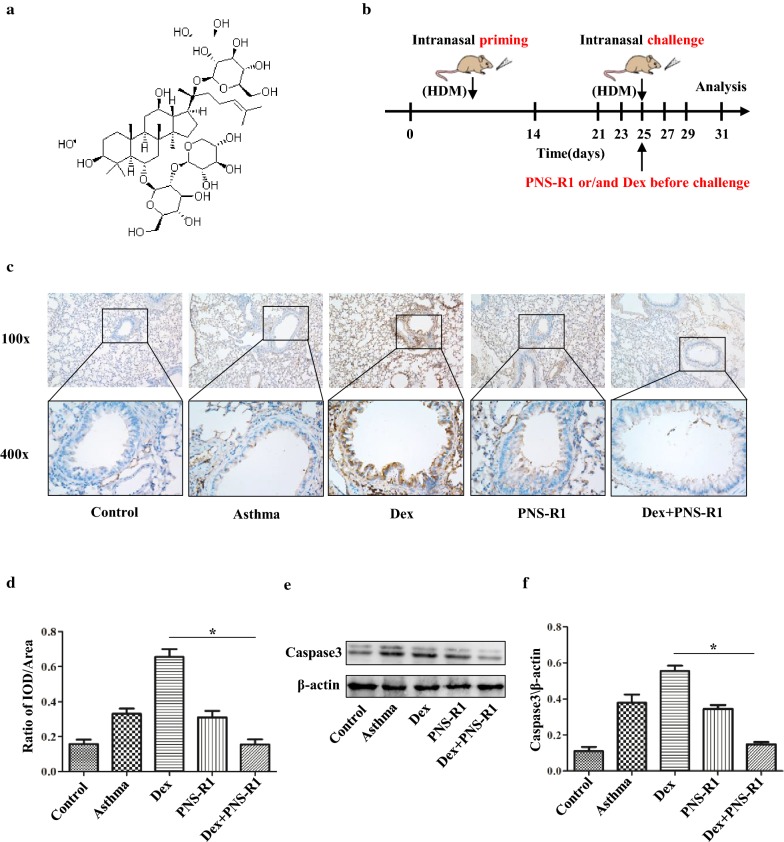
PNS-R1 reduces caspase3 expression of bronchial epithelial cells induced by Dex in asthmatic mice. **a** Chemical structure of PNS-R1 (C47H80O18, molecular weight = 933.13, purity > 98%). **b** Protocol for inducing asthmatic mice. **c** Immunohistochemical of caspase3 expression in bronchial epithelial cells (brown represents positive expression). **d** Statistics of immunohistochemical positive expression. **e** The protein expression of caspase3 in mouse lung tissue was determined by western blot analysis. **f** The results of the western blot were quantified. Data represent the average ± SD. Significant differences between two are indicated as *P < 0.05, Dex group vs Dex + PNS-R1 group

PNS-R1 is one of the extracts from traditional Chinese medicine pseudo-ginseng and its chemical formula is C47H80O18 (Fig. 1a). HDM was used to established asthma model as the protocol and we used Dex and PNS-R1to intervene in asthmatic mice (Fig. 1b). Caspase3 is an important protein involved in apoptosis. We performed immunohistochemical analysis and western blotting to investigate whether PNS-R1 or Dex can influence caspase3 expression in the lung tissues of mice. Results of immunohistochemical analysis showed caspase3 positive expression (brown) of lung tissues in Dex + PNS-R1 group is less than Dex group (P < 0.05, Fig. 1c, d). The result of western blot also revealed that caspase3 of Dex + PNS-R1 group is lower than Dex group (P < 0.05, Fig. 1e, f).

### PNS-R1 alleviates Dex-induced bronchial epithelial cells apoptosis in asthmatic mice

**Fig. 2:**
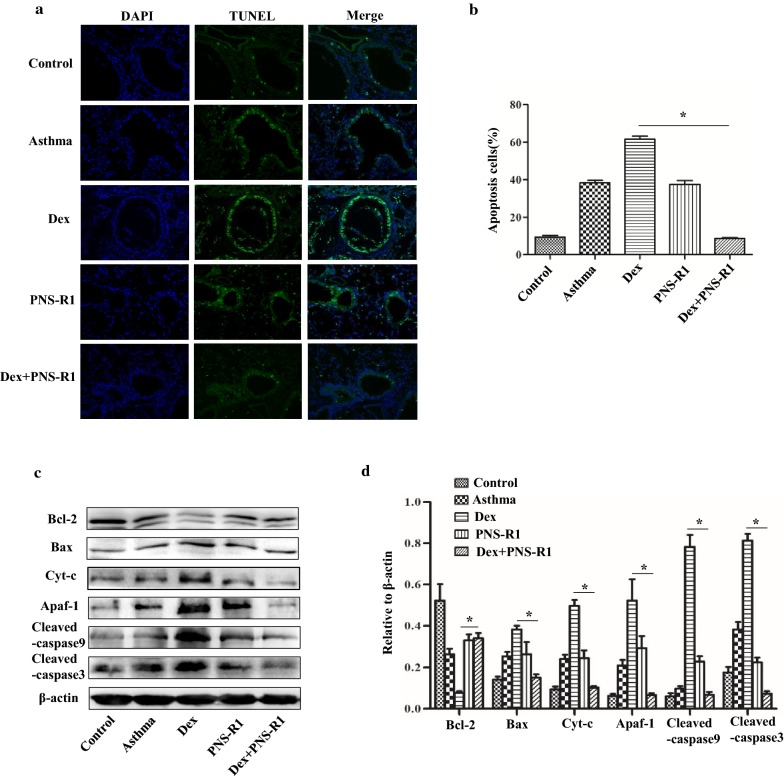
PNS-R1 decreases apoptosis of bronchial epithelial cells induced by Dex and inhibits the activation of mitochondrial apoptotic pathway in asthmatic mice. **a** The TUNEL assay revealed apoptosis of bronchial epithelial cells in different mice groups. **b** Statistical results of TUNEL positive cells percentage. Percentages of apoptosis (TUNEL-positive) cells were evaluated by using ImageJ software. Total number of cells was detected by DAPI. **c** Western blot was used for detection of mitochondrial apoptotic pathway including Bcl-2, Bax, Cyt-c, Apaf-1, cleaved-caspase3 and cleaved-caspase9 proteins. **d** The results of the western blot were quantified. Data represent the average ± SD. Significant differences between two are indicated as *P < 0.01, Dex group vs Dex + PNS-R1 group

To determine the anti-apoptotic effects of PNS-R1 in asthmatic mice, we used the TUNEL assay. Results of the TUNEL assay revealed that apoptotic cells of Dex + PNS-R1 group are less than Dex group (P < 0.05, Fig. 2a). Image-Pro Plus 6.0 software was used to measure the apoptotic cells in TUNEL assay and the apoptotic cells of Dex + PNS-R1 group are less than Dex group (P < 0.05, Fig. 2b).

### PNS-R1 inhibites the activation of mitochondrial apoptotic pathway in the asthmatic mice treated with Dex

Mitochondrial pathway is an important way to induce apoptosis, which includes Bcl-2, Bax, Cyt-c, Apaf-1 and other important proteins. Western blotting results showed that Bax, Cyt-c, Apaf-1, cleaved-caspase9 and cleaved-caspase3 expression in PNS-R1 + Dex group was downregulated relative to that in Dex group. Bcl-2 protein of PNS-R1 + Dex group was more than that of Dex group (Fig. 2c, d, P < 0.05). Collectively, these data suggest that PNS-R1 alleviates Dex-induced bronchial epithelial cells apoptosis partly by inhibiting mitochondrial apoptosis pathway.

### PNS-R1 attenuates Dex-induced 16HBE apoptosis

**Fig. 3:**
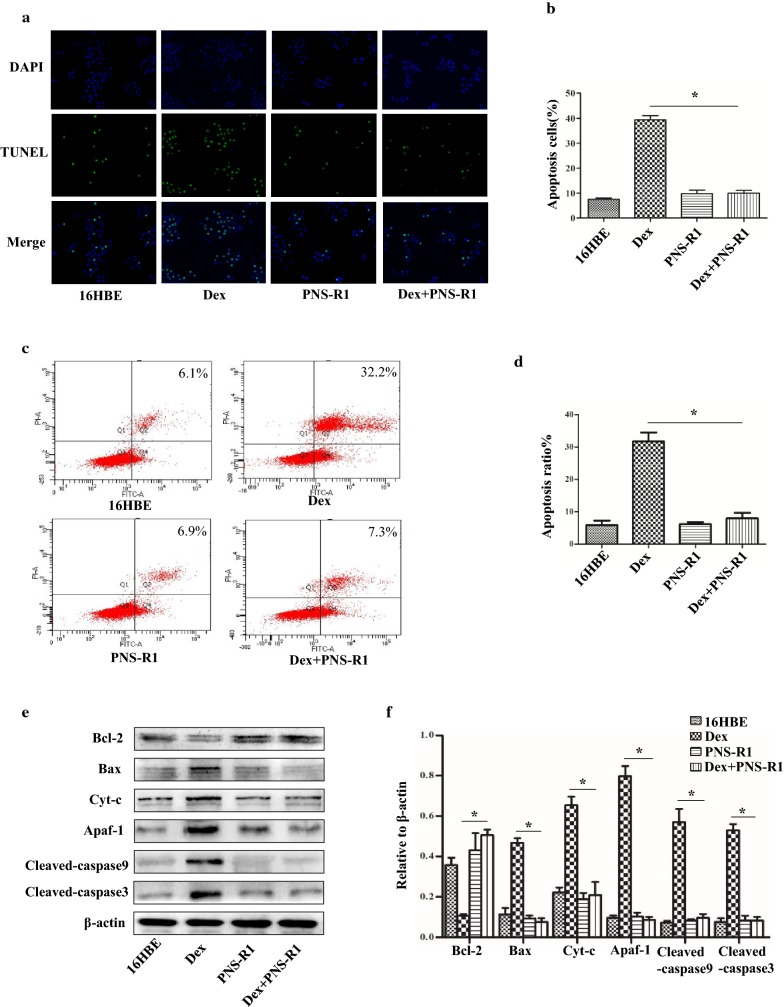
PNS-R1 attenuates Dex-induced apoptosis of 16HBE and inhibits the activation of mitochondrial apoptotic pathway. **a** The TUNEL assay was used to measure apoptosis of 16HBE. **b** Statistical results of TUNEL positive cells percentage. Percentages of apoptosis (TUNEL-positive) cells were evaluated by using ImageJ software. Total number of cells was detected by DAPI. **c** The Flow cytometry of Annexin V-PI apoptosis-detection kit revealed that apoptosis cells ratio of 16HBE. **d** Statistical results of cell apoptosis rate detected by flow cytometry. **e** Western blot was used to detect expressions of Bcl-2, Bax, Cyt-c, Apaf-1, cleaved-caspase3 and cleaved-caspase9 proteins in 16HBE cells. **f** The results of the western blot were quantified. Data represent the average ± SD. Significant differences between two are indicated as *P < 0.01, Dex group vs Dex + PNS-R1 group

In order to detect the protective effect of PNS-R1on apoptosis of bronchial epithelial cells induced by Dex, we used the human bronchial epithelial cell line-16HBE. 16HBE were treated by Dex or combined with PNS-R1 for 24 h. The TUNEL assay showed TUNEL-positive cells in Dex group were much more than Dex + PNS-R1 group and Image-Pro Plus 6.0 software was used for statistical analysis of the TUNEL-positive cells (Fig. 3a, b). Flow cytometry was used to measure the number of apoptotic cells (AV-positive). The results of flow cytometry revealed that apoptotic cells decreased in Dex + PNS-R1 group than the Dex group (P < 0.05, Fig. 3c, d). These results mean that PNS-R1 could obviously inhibit the apoptosis of bronchial epithelial cells induced by Dex.

### PNS-R1 protected against Dex-induced apoptosis in 16HBE by inhibiting the mitochondrial apoptosis pathway

**Fig. 4:**
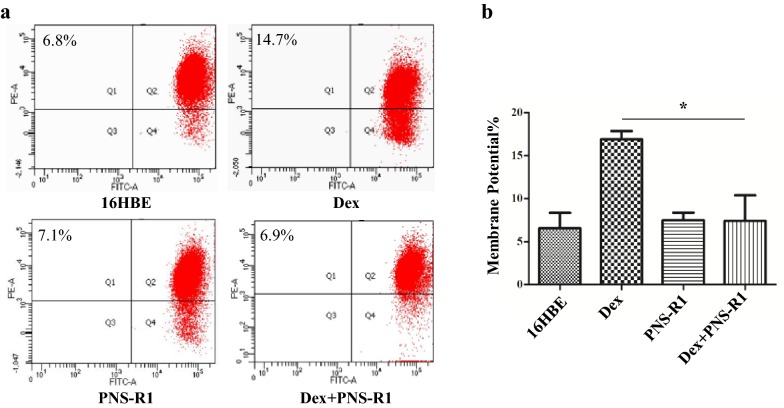
PNS-R1 reduces JC-1 positive cells in Dex treated 16HBE. **a** Flow cytometry was used to measure JC-1 of 16HBE. **b** Percentage of cells excited by a statistical flow pattern of green fluorescence. Data represent the average ± SD. Significant differences between two groups are indicated as *P < 0.01, Dex group vs Dex + PNS-R1 group

We have shown that PNS-R1 could influence mitochondrial apoptosis pathway in asthmatic mice treated with Dex. We next looked for the important proteins of mitochondrial apoptosis pathway in 16HBE. Western blot analysis showed that anti-apoptotic protein Bcl-2 was upregulated in Dex + PNS-R1 group compared with Dex group (P < 0.05). The Bax, Cyt-c, Apaf-1, cleaved-caspase9 and cleaved-caspase3 proteins expression of Dex + PNS-R1 group were less than Dex group (P < 0.05, Fig. 3e, f). The flow cytometry of JC-1 were performed to determine whether the mitochondrial pathway is involved in mediating the protective effects of PNS-R1 against Dex-induced apoptosis in 16HBE cells. Cells in the Dex + PNS-R1 group, which were exposed to Dex and PNS-R1 for 24 h, showed significantly less positive membrane potential than that of Dex group (P < 0.05, Fig. 4a, b). Taken together, we conclude that PNS-R1 might contribute to alleviate bronchial epithelial cells apoptosis induced by Dex through inhibiting mitochondrial apoptosis pathway.

## Discussion

Panax notoginseng saponins R1 (PNS-R1) is an important active ingredient of Panax notoginseng, which is an effective treatment for chronic inflammation diseases [15]. However, its bronchial epithelial cells properties and underlying mechanisms remain largely unknown. In the present study, a series of experiments were performed to determine whether PNS-R1 was able to ameliorate the apoptosis. A previous study suggested that PNS-Rb1 that can reduce cognitive and sensorimotor dysfunction by modulating the Akt/mTOR/PTEN signaling pathway [14]. In neonatal cerebral hypoxic-ischemic brain injury, notoginsenoside R1 was demonstrated to inhibit neuronal apoptosis and promote cell survival via the PI3K-Akt-mTOR/JNK signaling pathway [18]. PNS-R1 exerted protective effects against ischemia reperfusion (IR) injury in the heart by inhibiting the activation of the TGF-β1-TAK1 signaling pathway and attenuating apoptotic stress in the myocardium [19]. Pretreatment with NG-R1 suppressed the lipopolysaccharide (LPS)-induced degradation of inhibitor of nuclear factor-κB (NF-κB) α, the activation of NF-κB and caspase3, and the subsequent myocardial inflammatory and apoptotic responses in H9c2 cardiomyocytes [20]. Our current findings showed that treatment with PNS-R1 decreased apoptosis rates induced by Dex of bronchial epithelial cells. These results indicated that PNS-R1 also have protective effect on the apoptosis induced by Dex in the respiratory system.

It has been reported long-term use of GCs for the treatment of asthma has been associated with adverse side effects, thereby restricting their clinical application. Dex and other GCs are currently the most effective anti-inflammatory drugs available for the treatment of various chronic inflammatory and immune diseases, such as asthma [21, 22]. However, multiple studies indicated that Dex or other GCs can induce apoptosis in multiple cell types, such as hepatocytes, eosinophils, and chondrocytes [23, 24]. In addition, Dex exposure significantly increased the accumulation of apoptotic cells, caspase3 production, and TGF-β1/Smad2 pathway activity [25]. In our present study, we found that Dex could promote apoptosis of bronchial epithelial cells and increased Bax, Cyt-c, Apaf-1, caspase3, cleaved-caspase9 and cleaved-caspase3 expression, meanwhile decreased Bcl-2 expression. These datas showed that Dex induced apoptosis of bronchial epithelial cells partly affecting the mitochondrial apoptosis pathway. As demonstrated in previous studies, with the stimulation of pro-apoptosis factors, the Bax protein migrated from the cytoplasm to the outer mitochondrial membrane, changing the permeability of the outer mitochondrial membrane to promote the mitochondrial release of Cyt-c [26]. Release of Cyt-c leads to the formation of the apoptosome complex by the binding of Cyt-c to Apaf-1, procaspase9 and dATP. The dimer complex activates first caspase9 and then caspase3, and these activation results in apoptosis [27]. In the present study, we analyzed the changes of Bcl-2, Bax, Cyt-c, Apaf-1, cleaved-caspase9 and cleaved-caspase3 following treatment with PNS-R1 and Dex. Our findings have been found that PNS-R1 could decrease apoptosis of bronchial epithelial cells induced by Dex. PNS-R1 downregulated caspase3 expression in the lung tissues of Dex treated asthmatic mice and upregulated anti-apoptotic protein Bcl-2 expression. Treatment with PNS-R1, it could disrupt the mitochondrial membrane potential in Dex treated bronchial epithelial cells. PNS-R1 also reversed Dex-induced overexpression of Cyt-c, Apaf-1, cleaved-caspase9 and cleaved-caspase3 expressions in vivo and in vitro. These results suggest that PNS-R1 inhibited Dex-induced apoptosis of bronchial epithelial cells in asthma through mitochondrial apoptosis pathway.

## Conclusion

In summary, we showed that Dex could induce bronchial epithelial cells apoptosis in asthma. Our current results, for first time demonstrated that PNS-R1 alleviated Dex-induced apoptosis in bronchial epithelial cells via mitochondrial apoptosis pathway. These findings may lead to the development of PNS-R1 as an adjuvant drug for asthma. We are aware of the limits of this study, which assessed apoptosis without knockout the key proteins. Further research to study deeper molecular mechanisms. Moreover, PNS-R1 has a variety of pharmacological activities and biological targets; therefore, further study to identify the targets responsible for its pro-apoptotic effects is necessary.

## Abbreviations

PNS-R1: Panax notoginseng saponins R1
Dex: dexamethasone
Bcl-2: B-cell lymphoma-2
Bax: Bcl-2 associated X protein
Cyt-c: cytochrome C
Apaf-1: apoptotic protease activating factor-1
